# Effect of the 2022 COVID-19 booster vaccination campaign in people aged 50 years in England: Regression discontinuity analysis in OpenSAFELY-TPP

**DOI:** 10.1016/j.vaccine.2025.127257

**Published:** 2025-05-20

**Authors:** Andrea L. Schaffer, William J. Hulme, Elsie Horne, Edward P.K. Parker, Venexia Walker, Catherine Stables, Amir Mehrkar, Seb C.J. Bacon, Chris Bates, Ben Goldacre, Alex J. Walker, The OpenSAFELY Collaborative, Miguel A. Hernán, Jonathan A.C. Sterne

**Affiliations:** a Bennett Institute for Applied Data Science, Nuffield Department of Primary Care Health Sciences, University of Oxford, Oxford, UK; b Population Health Sciences, Bristol Medical School, University of Bristol, Bristol, UK; c National Institute of Health and Care Research, Bristol Biomedical Research Centre, Bristol, UK; d London School of Hygiene & Tropical Medicine, London, UK; e National Institute for Health and Care Research ( NIHR) Health Protection Research Unit in Vaccines and Immunisation, London, UK; f Department of Surgery, Perelman School of Medicine, University of Pennsylvania, Philadelphia, USA; g TPP, TPP House, Leeds, UK; h CAUSALab, Harvard T.H. Chan School of Public Health, Boston, USA; i Departments of Epidemiology and Biostatistics, Harvard T.H. Chan School of Public Health, Boston, USA; j Health Data Research UK South-West, Bristol, UK

**Keywords:** Covid-19, Vaccination, Sars-cov-2, Influenza

## Abstract

SARS-CoV-2 vaccines are highly effective in preventing severe COVID-19 but require boosting to maintain protection. Changes to circulating variants and prevalent natural immunity may impact on real-world effectiveness of boosters. With NHS England approval, we used linked routine clinical data from >24 million patients to evaluate the effectiveness of the 2022 combined COVID-19 autumn booster and influenza vaccine campaign in non-clinically vulnerable 50-year-olds in England using a regression discontinuity design. Our primary outcome was a composite of 6-week COVID-19 emergency attendance, COVID-19 unplanned hospitalisation, or death. By 26 November 2022, booster vaccine coverage was 11.1 % at age 49.75 years increasing to 39.7 % at age 50.25 years. The estimated effect of the campaign on the risk of the primary outcome in 50-year-olds during weeks 7–12 after the start of the campaign was −0.4 per 100,000 (95 % CI -7.8, 7.1). The results were similar when using different follow-up start dates or when estimating the effect of vaccination (rather than the campaign). This study found little evidence that the autumn 2022 vaccination campaign in England was associated with a reduction in severe COVID-19-related outcomes among non-clinically vulnerable 50-year-olds. Possible explanations include the low risk of severe outcomes and substantial pre-existing vaccine- and infection-induced immunity. The booster campaign may have had effects beyond those we estimated, including reducing virus transmission and incidence of mild or moderate COVID-19.

## 1 Introduction

The SARS-CoV-2 vaccines are highly effective at preventing severe COVID-19 outcomes, including hospitalisation and mortality. [1,2] However, their benefits wane over time [3] and so booster vaccinations are needed to sustain protection. A first booster vaccination reduces the incidence of severe COVID-19, but that protection also wanes and may be reduced against new variants. [4–9] High prevalence of immunity resulting from prior SARS-CoV-2 infection may also impact on booster vaccine effectiveness.

Following the primary course of two doses, the UK offered first COVID-19 booster vaccinations from autumn 2021 to high-risk individuals and people 50 years and older. A spring 2022 booster was subsequently offered to people 75 years or older and those considered clinically vulnerable. An autumn booster was available from September 2022, initially targeting people considered at high risk, such as those aged over 65 years, care home residents and their staff, and immuno-suppressed people. [10,11] On 15 October 2022, people aged 50–64 years who were not considered high risk became eligible for booster vaccination. [12] This coincided with the 2022/23 rollout of influenza vaccination, which was available from the same date to people who would turn 50 by March 2023. [12]

In this study, we estimated the effectiveness of the 2022 autumn COVID-19 booster vaccination campaign, coinciding with the influenza vaccination campaign, in reducing COVID-19 outcomes among non-clinically vulnerable people aged 50 years in England using a regression discontinuity design and the OpenSAFELY-TPP database.

## 2 Materials and methods

### 2.1 Study objective and population

Four COVID-19 booster vaccines were offered to adults in autumn 2022, including two Moderna mRNA (monovalent and bivalent) and two Pfizer-BioNTech vaccines (monovalent and bivalent). For influenza, costs of the cell-based and recombinant quadrivalent influenza vaccines were reimbursed through the NHS for people under 65 years.

Our primary objective was to estimate the average effect of the combined COVID-19 booster and influenza vaccination campaign on severe COVID-19 outcomes in non-clinically vulnerable people aged 50 years in England who had previously received at least two COVID-19 vaccinations. This population became eligible for the COVID-19 booster and influenza vaccine on 15 October 2022. Our secondary objective was to estimate the effect of the booster vaccine itself among compliers; that is, people who take up the vaccine only when eligible.

We included all adults aged 45–54 years during the study period who were registered at one GP practice for at least 90 days prior to 3 September 2022 and had complete information on age and sex. People considered high risk or clinically vulnerable, who were eligible for booster vaccination earlier in the year were excluded. [10] This included people who [13]: identified as a health or social care workers at time of prior vaccination; were resident in a care or nursing home; or were part of any other clinically vulnerable group, specifically people with: chronic respiratory disease, chronic heart/vascular disease, chronic kidney disease, chronic liver disease, chronic neurological disease, diabetes, immunosuppression, asplenia, morbid obesity, or severe mental illness; or had evidence of having received a third primary dose of the COVID-19 vaccine which may be a marker of immunosuppression.

We also excluded people who were otherwise ineligible for the booster or unlikely to be vaccinated, including people who: received another COVID-19 vaccine within 90 days prior to 15 October 2022; did not receive the first two primary doses of the COVID-19 vaccine; were housebound; or were receiving end of life care. Supplementary Table 1 lists the exclusion criteria and their definitions. Clinically vulnerable individuals were identified using primary care data using the same approach as described previously. [3,14]

### 2.2 Data source

All data were linked, stored and analysed securely within the OpenSAFELY platform: https://opensafely.org/. With the approval of NHS England, primary care records managed by the GP software provider TPP SystmOne were linked, using NHS numbers, to Emergency Care Data Set (ECDS) and in-patient hospital spell records via NHS Digital’s Hospital Episode Statistics (HES), and national death registry records from the Office for National Statistics (ONS). COVID-19 vaccination history is available in the GP record directly via the National Immunisation Management System (NIMS). The dataset analysed within OpenSAFELY is based on approximately 24 million people currently registered with GP surgeries using TPP SystmOne software.

### 2.3 Study measures

#### 2.3.1 Outcomes

The primary outcome was a composite of COVID-19-related unplanned hospitalisation, COVID-19-related accident and emergency attendance, or COVID-19 death from 6-weeks after start of the vaccination campaign. If an individual had multiple events during the followup period, it was only counted once. For unplanned hospitalisations, the COVID-19 diagnosis could be either primary or secondary cause in any position. Only unplanned admissions were included as these are more likely to be due to incident COVID-19 disease. For COVID-19 deaths, COVID-19 could be either an underlying or contributing cause in any position. We did not include a positive SARS-CoV-2 test as an outcome because free testing in England ended in April 2022.

Due to potential misdiagnosis of COVID-19 related outcomes, we also included a composite outcome of respiratory unplanned admission or respiratory death. We also examined any unplanned hospitalisations and any death. The definition and codes used to identify outcomes are in Supplementary Table 2.

#### 2.3.2 Exposure

The exposure was booster eligibility, defined as being 50 years or older on the start of follow-up, for vaccination in the autumn 2022 booster campaign. Age was categorised in 3-month intervals. As only month of birth is available in OpenSAFELY, date of birth was set to the 15th of the month. We defined receipt of the autumn booster as a record of a third or fourth COVID-19 vaccination on or after 5 September 2022 (the date autumn boosters first became available), as some people may have been vaccinated prior to eligibility. We constructed cumulative incidence curves of booster coverage to display how separation above/ below the threshold changed over the study period.

### 2.4 Study design

We used regression discontinuity, a study design that takes advantage of the threshold of being aged 50 years or older for booster vaccination eligibility and estimates the effectiveness of booster vaccination at this threshold. [15,16] Threshold-based eligibility mimics randomisation, as the distribution of confounding variables among people just above and below the threshold is expected to be similar. [17] Regression discontinuity has previously been used to estimate the effectiveness of vaccines and vaccination campaigns, such as the first COVID-19 vaccine dose on COVID-19 mortality in England [18] and influenza vaccination in England and Wales. [19]

**Fig. 1:**
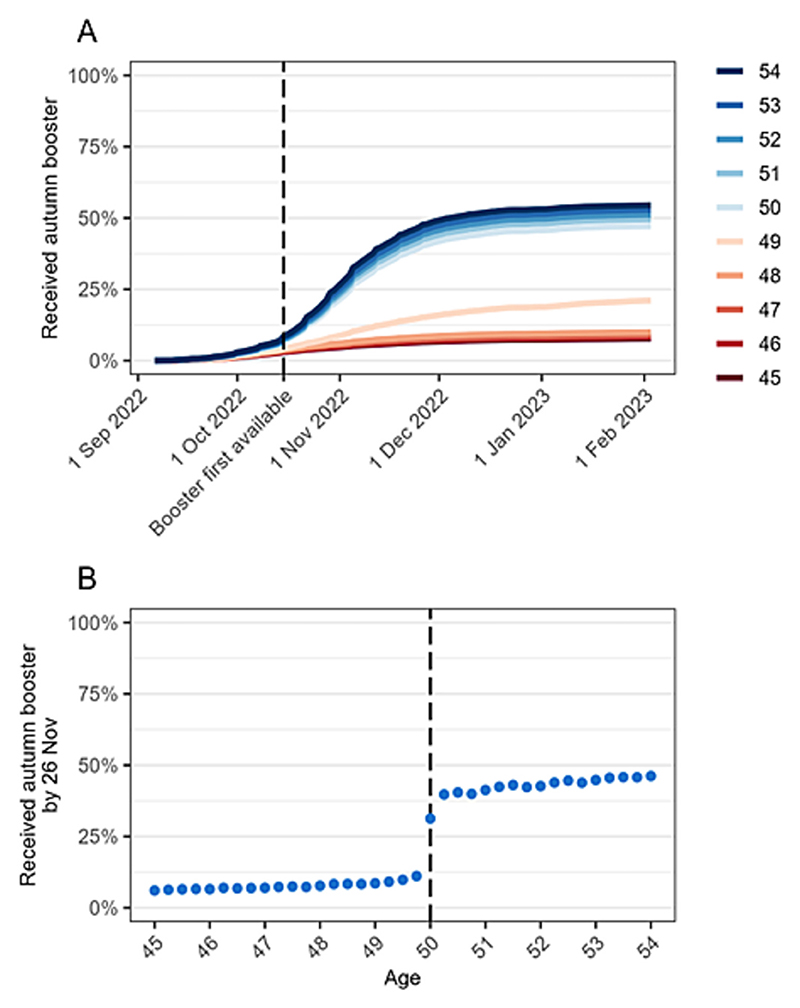
Coverage (%) of COVID-19 autumn booster by age. A) Cumulative coverage based on age in years at 3 September 2022. The booster vaccination became available to non-clinically vulnerable people aged 50–64 years on 15 October 2022. B) Coverage at 26 November 2022 by age in 3-month intervals.

For the primary analysis, the index date for start of follow-up was 6 weeks after the start of the campaign (26 November); this was chosen as booster vaccine coverage started to plateau at this point (Fig. 1a). The population-level effect of booster vaccination is likely to evolve over time, as the proportion of people vaccinated increases, and the prevalence of SARS-CoV-2 infection changes. We therefore included multiple index dates for the start of follow-up as supplementary analyses: 3 September 2022 (before the start of the campaign; negative control); 15 October 2022 (start of the campaign; negative control); and each day between 26 November (6 weeks after the start of the campaign) and 9 December 2022. Follow-up was for 6 weeks after each index date. For example, for the analysis starting on 9 December we identified outcomes up to 20 January. For each index date, we excluded people who had died or deregistered before that date.

#### 2.4.1 Assumptions

Key assumptions of regression discontinuity are “continuity” (the risk of the outcome is expected to change smoothly at the threshold in absence of the intervention) and “exchangeability” (similar distribution of confounders just below and above the threshold). [17] To test the latter assumption, we plotted the distribution of the following characteristics by age in 3 month intervals: sex (male, female); deprivation, measured by the English Index of Multiple Deprivation (IMD), grouped by quintile of national rank; ethnicity (White, Mixed, Asian/Asian British, Black/Black British, Other, Unknown); practice region (East, East Midlands, London, North East, North West, South East, South West, Yorkshire and The Humber); number of previous COVID-19 vaccine doses. We also quantified receipt of influenza vaccine in the 2022/23 season from July 2022 onwards to examine its discontinuity at the threshold. However, ascertainment of influenza vaccination was incomplete, because vaccination delivered by pharmacists outside of general practice is not routinely recorded in electronic health records.

We used both “sharp” and “fuzzy” regression discontinuity designs, which address different research questions. [16,20] The sharp design estimates the effect of the vaccination campaign (rather than receipt of vaccination itself) at the threshold age. As both the second booster and the influenza vaccine became available on the same date at the same age threshold, this represents the effect of the combined vaccination campaigns. In contrast to the sharp design, the fuzzy design estimates the effectiveness of receiving, compared with not receiving, booster vaccination. Fuzzy regression discontinuity uses vaccine eligibility (being age 50 years or older) as an instrumental variable and estimates the “local average treatment effect” (LATE) or “complier average causal effect” (CACE) at the threshold. [17] This is the effect of vaccination among “compliers” - the subset of the population who are vaccinated only when eligible. The analysis assumes no “defiers” - people who are vaccinated only when ineligible. This assumption is also known as monotonicity.

The monotonicity assumption is untestable, but likely to hold in our study. Other assumptions are that the instrumental variable is associated with the outcome only through vaccination at the threshold, and there are no common causes of eligibility and the outcome. [21] These assumptions are also plausible for our study.

### 2.5 Statistical methods

Outcomes were expressed as 6-week risks per 100,000 population. To prevent disclosure, all event counts presented were rounded to the nearest 5 with risks calculated from rounded counts. However, unrounded counts and risks were used in regression modelling. The primary bandwidth was 5 years (20 data points representing three-month age intervals) either side of the threshold.

To estimate the effect of being eligible for the COVID-19 autumn booster/influenza vaccine among people aged 50 years (sharp regression discontinuity), we estimated the discontinuity in the risk of each outcome at the threshold by fitting a regression model with age in 3-month intervals (continuous), a binary variable representing the vaccine age threshold (≥50 years), and an interaction between the two, allowing the slope of the change in the probability of the outcome by age to vary above and below the threshold. Age was centred, so that “0” represented the threshold. We expressed estimated effects as risk differences with associated 95 % confidence intervals (CIs). This represents the estimated difference in the 6-week outcome risk among people just above the threshold (who were targeted by the campaign) compared with people just below the threshold (who were not targeted by the campaign). A negative value means that the vaccination campaign is associated with a lower rate of the outcome. For the fuzzy regression discontinuity analysis, we used an instrumental variable approach to estimate the effect of vaccination in people aged 50. We used the two-stage least squares estimator. As is standard for instrumental variable analysis, we first predicted booster vaccine coverage based on age. We then predicted the outcome using the age-based predictions of vaccine coverage. [17] Here, the risk difference represents the estimated difference in the 6-week outcome among “compliers”, that is the subset of the population who are vaccinated only when eligible.

### 2.6 Sensitivity analyses

For the sharp regression discontinuity analysis, we conducted two sensitivity analyses: first, we excluded people born in the index month due to the imprecision in recorded birth date. Second, given the potential for bias with wide bandwidths, we repeated the analysis using progressively smaller bandwidths (4 years, 3 years, 2 years, 1 year). For the fuzzy regression discontinuity analysis, we repeated the analysis including influenza vaccination prior to the index date in the model and using different bandwidth periods.

### 2.7 Software and reproducibility

Data management was performed using Python 3.8, with analysis carried out using R 4.0.5. Code for data management and analysis as well as codelists archived online https://github.com/opensafely/vax-fourth-dose-RD.

### 2.8 Patient and Public Involvement

We have developed a publicly available website https://opensafely.org/ through which we invite any patient or member of the public to contact us regarding this study or the broader OpenSAFELY project.

### 2.9 Ethics approval

This study was approved by the Health Research Authority (Research Ethics Committee.

Reference 20/LO/0651) and by the London School of Hygiene and Tropical Medicine ethics

Board (reference 21863).

## 3 Results

We identified 3,254,000 people aged 45–54 years registered with a practice using TPP SystmOne on 3 September 2022. Of these, 1,336,625 (41.1 %) were excluded (Table 1). The most common reasons for exclusion were not having received a second primary COVID-19 dose (521,165, 16.0 %) or being in a clinically vulnerable group (762,650, 23.4 %), most frequently due to diabetes (229,905, 7.1 %), chronic heart disease (160,165, 4.9 %) or severe obesity (143,720, 4.4 %).

Among 1,917,375 people included in analyses, 465,730 (51.4 %) of people 45–49 years and 516,310 (51.1 %) of people 50–54 years were male and 661,145 (73.0 %) and 776,560 (76.8 %) were of White ethnicity, respectively (Table 2). Most (743,485 [82.1 %] and 885,095 [87.5 %]) had received three previous COVID-19 vaccine doses. The median time since the previous dose at the start of the campaign (15 October) was 304 days (interquartile range [IQR], 297–321 days) and 310 days (IQR, 301–328), respectively.

### 3.1 Vaccination coverage

A small number of people in our study population received the booster vaccine prior to its wider availability on 15 October 2022 (Fig. 1A). Coverage increased rapidly thereafter and started to plateau by late November. Six weeks after the wider availability (26 November), booster coverage ranged from 6.1 % of people 45.00 years to 51.8 % of people aged 54.75 years (Fig. 1B). A large discontinuity was observed at the threshold, with the proportion receiving booster vaccination increasing from 11.1 % of people aged 49.75 years to 31.1 % and 39.7 % of people aged 50.00 and 50.25 years respectively.

No discontinuity was observed for any of the demographic variables (sex, IMD quintile, ethnicity, region) (Supplementary Figs. 1–4) or number of prior COVID-19 vaccine doses (Supplementary Fig. 5). This suggests that the assumption of exchangeability (no discontinuity in confounders at the threshold) was met for these factors. However, we observed a discontinuity in recorded receipt of the 2022/23 influenza vaccine, increasing from 9.7 % of people aged 49.25 years to 25.5 % of people aged 50.00 years (Supplementary Fig. 6). Recorded receipt of influenza vaccine was much more common among people who received COVID-19 booster vaccination. In people aged 45–49 years, 36,515 (53.2 %) of those receiving booster vaccination also received influenza vaccination, compared with 46,820 (5.6 %) of those who did not receive booster vaccination. Corresponding figures in people aged 50–54 years were 277,745 (64.7 %) and 68,340 (11.8 %).

### 3.2 Regression discontinuity analysis

Overall, the COVID-19 composite outcome (COVID-19 unplanned admission, COVID-19 A&E attendance, or COVID-19 death) was rare; the risks within 6 weeks of 26 November 2022 were 12.7 and 14.4 per 100,000 for people aged 45–49 and 50–54 years respectively (Table 3). During the same period, the 6-week risks of the respiratory composite (unplanned respiratory admission or respiratory death) were 52.0 and 53.5 per 100,000 respectively, any unplanned admission 410.5 and 447.0 per 100,000 respectively, and any death 6.1 and 13.4 per 100,000 respectively.

**Fig. 2:**
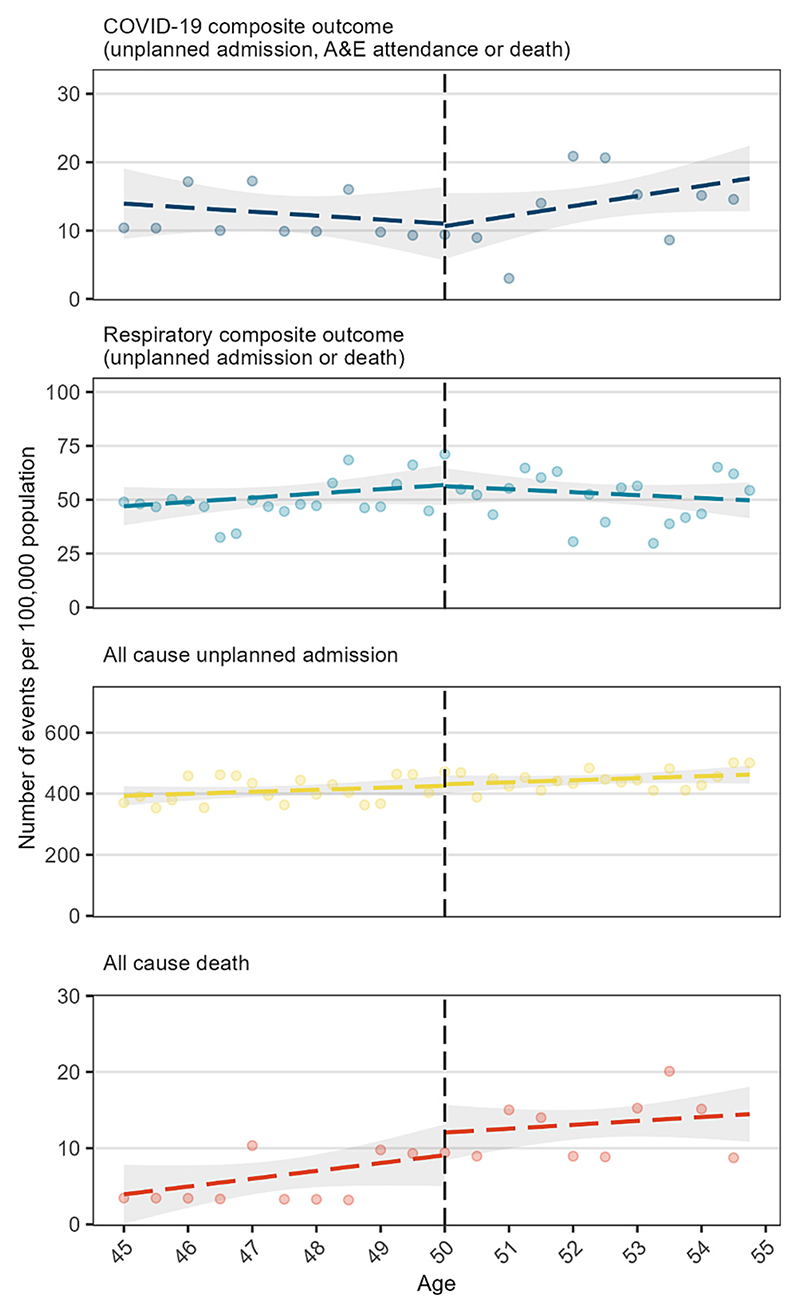
Predicted values (dashed line) and 95 % confidence interval (shaded area) from sharp regression discontinuity analysis of 6-week outcomes by age in 3-month intervals with 26 November as the index date. The dots are the observed values and should be considered approximations only; to prevent disclosure, rates are calculated from rounded counts and for the COVID-19 composite outcome and any death outcome, observed values for 6-month intervals are presented instead of 3 months for visualisation only. Predicted values were estimated using unrounded counts.

**Fig. 3:**
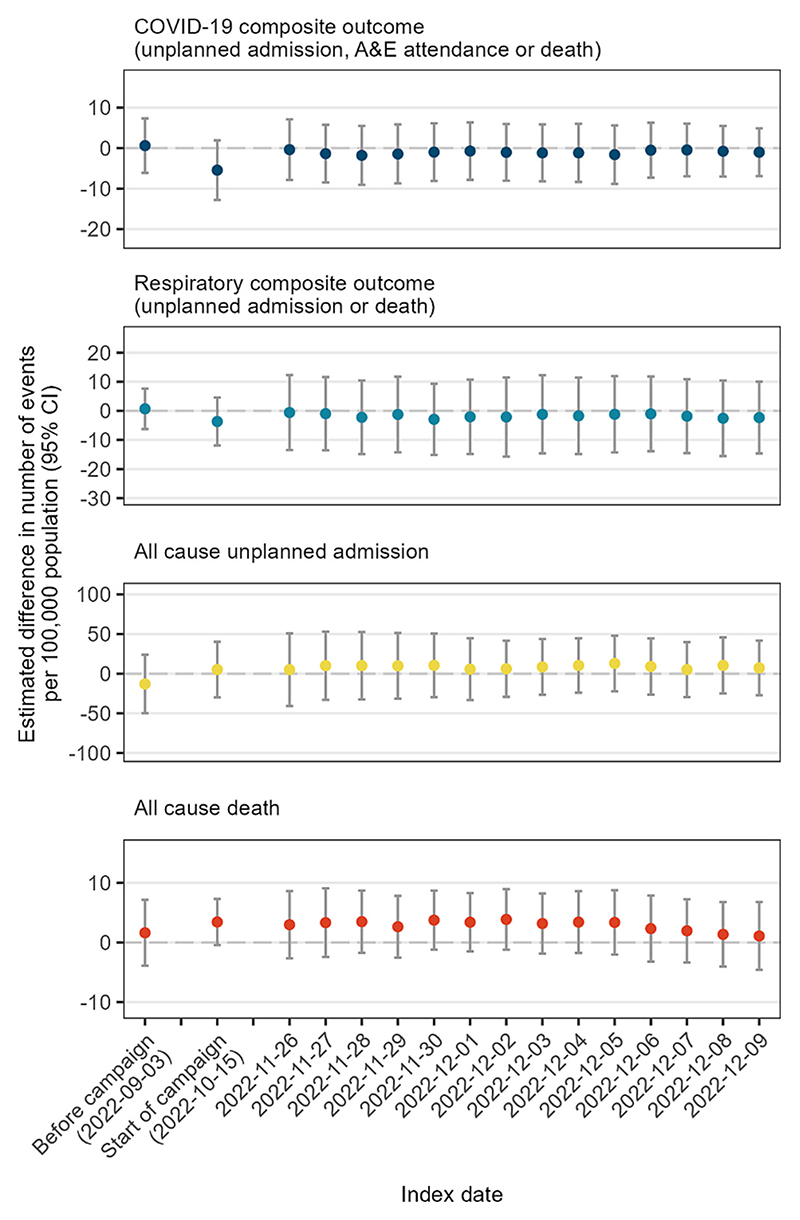
Estimated difference in 6-week outcome risk per 100,000 among people aged 50 years using different index dates. A positive estimate indicates a higher risk among people eligible to receive the booster vaccination compared with those ineligible. Whiskers represent 95 % confidence intervals.

By age, during the six weeks from 26 November 2022 the risk of the COVID-19 composite outcome was relatively constant between ages 45 and 55 years, with a slight negative slope before age 50 and a slight positive slope thereafter (Fig. 2). The estimated effect of the booster vaccination campaign in 50-year-olds on the 6-week risk was −0.4 per 100,000 (95 % CI -7.8 to 7.1), with a negative value indicating a slightly lower risk among people who were eligible for the campaign (Table 4). Similarly, for the respiratory composite outcome the estimated effect on 6-week risk −0.6 per 100,000 (95 % CI -13.5 to 12.3) and for any unplanned admission 5.0 per 100,000 (95 % CI -40.7 to 50.8). For any death the effect was 3.0 per 100,000 (95 % CI -2.7 to 8.6). Fig. 3 shows corresponding estimates of the effect of the booster campaign for each index date and each outcome for people at the threshold: the estimates for index date 26 November correspond to those shown in Fig. 2. Results were similar using different index dates for all outcomes (Table 4). These results also were little changed after excluding people born in the index month (Supplementary Table 3). The results were robust to different bandwidths, but as expected the confidence intervals were wider for shorter bandwidths (Supplementary Table 4).

The instrumental variable analysis (fuzzy regression discontinuity) estimates the effectiveness of the booster vaccine in compliers at the threshold. Based on the first stage of the instrumental variable analysis, the estimated proportion of compliers among the population at the threshold is 28 %. This analysis found a difference of 2.1 per 100,000 compliers for the COVID-19 composite outcome (95 %CI -11.3 to 15.4) (Supplementary Table 5). This is expected given the absence of evidence for a population-level effect of the booster vaccination campaign. Controlling for prior receipt of influenza vaccination did not change the conclusions of the instrumental variable analyses, although confidence intervals became wider (Supplementary Table 6). Similarly, using different bandwidths did not change the findings (Supplementary Table 7).

## 4 Discussion

### 4.1 Summary

In this study of over 1.9 million people aged 45–54 years in England, there was a low risk of severe COVID-19 outcomes among non-clinically vulnerable people aged 45–54 years who had received at least two COVID-19 vaccine doses, and only moderate coverage of booster vaccination in people aged ≥50 years in autumn/winter 2022/23. Using a regression discontinuity design, we found little evidence of a population effect of the combined COVID-19 booster and influenza vaccination campaign on severe COVID-19 related events, unplanned respiratory or all-cause admissions or death in people aged 50 years. Secondary analyses of receipt of the vaccine (fuzzy regression discontinuity) also found no evidence of an effect. However, confidence intervals for the fuzzy analysis were wide.

### 4.2 Findings in context

Various studies worldwide have demonstrated the effectiveness of a second booster against SARS-CoV-2 infection or severe COVID-19 outcomes when the Omicron variant predominates, with many identifying rapid waning. [22–24] However, most studies focussed on older populations (60+ years), or do not report age-specific effectiveness estimates. Therefore, there are few studies with which to compare our findings.

Among the full population (no age restriction), a study using a Susceptible-Exposed-Infectious-Recovered model estimated that the UK booster campaign resulted in approximately 19,000 fewer hospitalisations and 1500 fewer deaths compared with a no booster scenario. [25] Another study evaluated the 2022 autumn booster vaccination campaign in England using a test-negative design: this found that the effectiveness of three or more COVID-19 vaccine doses against COVID-19 hospitalisation in people 18–64 years was 61.5 % compared with unvaccinated people in the first two weeks post-vaccination, falling to 38.9 % by 6 months. [26] Additionally, the incremental effectiveness of the booster against hospitalisation among people 50+ years who had previously received at least two doses was estimated to be 43.7 % and 47.5 % at 5–9 weeks post-vaccination for the Pfizer and Moderna boosters respectively.

A few studies compared receipt of a fourth dose to third dose only, although the resulting estimates will be influenced by the dominant strain and pre-existing immunity in the population. A study in Nordic countries found reductions in hospitalisation and death of −28.0 % (95 %CI 5.6 % to 50.3 %) and 8.7 % (−67.8 % to 85.3 %) respectively, among people 50–69 years who received a fourth dose. [27] A study of people aged ≥18 years in Singapore found that a fourth bivalent dose provided additional protection against COVID-19 hospitalisation among both SARS-CoV-2 naïve (HR = 0.12, 95 %CI 0.08–0.18) and non-naïve (HR = 0.04, 95 %CI 0.01–0.15) people. [28]

By contrast with these studies, our analysis evaluated the impact of the vaccination campaign in non-clinically vulnerable people aged 50 years, whereas effectiveness studies using other designs focus on the effect of receipt of vaccination. Therefore, these studies should be viewed as complementary, rather than directly comparable.

### 4.3 Policy implications and interpretation

Our study included nearly 2,000,000 people, and the estimated effect of the booster vaccination campaign on the 6-week risk of our primary outcome in 50-year-olds was –0.4 per 100,000 (95 %CI -7.8 to 7.1). This is equivalent to a difference of 0.0004 % (95 %CI -0.0008 % to 0.0007 %), a confidence interval that excludes any important effect of the booster campaign. The effect of the vaccination campaign must be considered in the context in which they are implemented, due to changes in circulating variants, prevalence of previous infections, and changes in vaccine coverage. Our study was conducted during a time of high substantial pre-existing immunity. All participants had received at least two COVID-19 vaccine doses, with 85 % having received three prior doses, and natural infection during successive omicron waves in 2022 was widespread. We previously estimated absolute vaccine effectiveness (compared with unvaccinated individuals) against COVID-19 hospitalisation of >80 % at 6 months after primary vaccination with ChAdOx1-S among individuals aged 40–64 years in England, and this protection would have been enhanced among individuals who received a vaccine booster dose. [3] The resulting high baseline immunity in our study population may have reduced the additional effectiveness of the autumn 2022 booster campaign.

The autumn 2022 booster campaign in England was initiated to mitigate a probable winter COVID-19 wave. Vaccination campaigns are planned based on the best available knowledge at the time and cannot necessarily anticipate how widespread the wave will be. During our study period, the estimated prevalence of SARS-CoV-2 infection was between 2 % and 3 % among people 35–69 years, meaning that the risk of severe COVID-19 was low. [29] Our study estimated the impact of the autumn booster campaign in 50-year-olds who were not clinically vulnerable, a group already at low risk of severe COVID-19. Vaccination coverage in people eligible for the vaccine was modest, ranging from 40 % at age 50.25 years to 52 % at age 54.75 years. This will have limited our ability to identify effects of booster vaccination, because differences between people just above and below the age threshold will be attenuated compared with a direct comparison of the effects of vaccination with no vaccination.

This study focussed on severe outcomes, but there are potential benefits of vaccinating low-risk populations that we were not able to examine. Illness that does not result in hospitalisation or death can still lead to loss of productivity and potentially long-term post-acute sequelae of COVID-19. [30,31] We could not evaluate the impact of the campaign on mild COVID-19 or SARS-CoV-2 infections as these are not well-captured in electronic health records since the cessation of free testing. [32] We could not also evaluate indirect effects of booster vaccination. Public health campaigns can have spillover effects [33] extending beyond the targeted population, and vaccination of non-high-risk individuals can benefit the rest of the community by limiting virus transmission. These findings may also not hold in future waves, with different SARS-CoV-2 transmissibility, circulating variants, and infection prevalence.

While COVID-19 booster vaccinations are extremely safe, [34] no pharmacological intervention is without risk. The relative risks and benefits of health interventions need to be considered, and the balance is likely to shift over time as the pandemic matures. For instance, in March 2023 the WHO Strategic Advisory Group of Experts on Immunisation updated their guidance and does not recommend second boosters for non-high risk individuals, which includes healthy people aged <60 years. [35] Our findings cannot be generalised to older people (because we estimated the effect of booster vaccination at age 50 years) or to those who are clinically vulnerable (because they were excluded from the population analysed). Booster vaccine effectiveness during autumn 2022 may have been higher in older adults, as was reported for booster doses administered in autumn 2021. [14] However, there may be a case for changing eligibility thresholds in future campaigns, especially if vaccine costs and availability are a consideration, and there are expectations of low vaccine coverage. Continued monitoring of vaccination campaigns for emerging signals is warranted.

### 4.4 Strengths and weaknesses

The OpenSAFELY-TPP database covers approximately 40 % of registered English primary care patients [36] enabling analysis of a large cohort of people aged within five years of the booster vaccination age eligibility threshold. These data include reliable ascertainment of COVID-19 vaccination status, COVID-19-related outcomes, and other clinical characteristics.

Establishing causality using observational data is challenging. [37] Unlike many observational study designs, regression discontinuity an-alyses are unlikely to be impacted by unmeasured confounding. Vaccination campaigns are well-suited to regression discontinuity designs, as eligibility is often based on a threshold, usually age. There is a reasonable assumption of “exchangeability” of people on either side of this threshold. We checked this assumption and did not find discontinuities for several potentially confounding variables (e.g. deprivation), a notable exception being receipt of influenza vaccination which was subject to the same age eligibility threshold on the same date. It is possible that other, unmeasured, confounders did exhibit discontinuity at the threshold, but we are not aware of any other factor that could reasonably be expected to differ substantially between 49 year olds and 50 year olds. If receipt of the influenza vaccine was a confounder, we would expect it to increase the apparent effectiveness of the COVID-19 vaccination campaign by reducing the risk of respiratory outcomes, which was not observed. Regardless, our results should be considered to estimate the combined effect of the COVID-19 booster and influenza vaccination campaigns at age 50 years as we cannot disentangle the effects of the two vaccines.

In addition to estimating the effect of the campaign, we also used a fuzzy regression discontinuity design to estimate the effectiveness of the vaccine itself. However, this analysis has limited precision leading to wide confidence intervals which do not exclude a large relative effect of vaccination. Our analysis also does not account for non-independence within and across exposure groups, arising from transmissibility of SARS-CoV-2. People below age 50 may indirectly benefit from booster vaccination among their slightly older peers due to reduced transmission rates, resulting in lower estimates of effectiveness in the sharp discontinuity design.

## 5 Conclusion

In this study, we found little evidence that the autumn 2022 COVID-19 booster vaccination campaign (coinciding with the influenza vaccination campaign) in England reduced severe COVID-19-related outcomes among non-clinically vulnerable 50-year-olds who had received at least two previous vaccination doses. Possible explanations include the low risk of severe outcomes due to substantial pre-existing vaccine- and infection-induced immunity. Modest booster coverage reduced the precision with which we could estimate effectiveness. The booster campaign may have had effects beyond those estimated, including reducing virus transmission and incidence of mild or moderate COVID-19.

## Supplementary Material

Supplementary data to this article can be found online at https://doi.org/10.1016/j.vaccine.2025.127257.

Supplementary material

## Figures and Tables

**Table 1:** All people 45–54 years on 3 September 2022 registered with a general practice using TPP SystmOne software and reasons for exclusion from the study. People could have multiple exclusion criteria and so may appear in multiple categories. All counts rounded to nearest 5.

	N (%)^
**Total prior to exclusions**	3,254,000
	(100.0)
**Any exclusion**	1,336,625 (41.1)
**Included in final cohort**	1,917,375 (58.9)
**Prioritised for vaccination**	
Living in care home	7595 (0.2)
Health and social care worker	149,070 (4.6)
Clinically vulnerable	762,650 (23.4)
Immunosuppressed	99,370 (3.1)
Chronic kidney disease	29,760 (0.9)
Chronic respiratory disease	60,345 (1.9)
Asthma	14,440 (0.4)
Diabetes	229,905 (7.1)
Asplenia	19,130 (0.6)
Chronic liver disease	92,525 (2.8)
Chronic heart disease	160,165 (4.9)
Severe mental illness	41,680 (1.3)
Severe obesity (BMI ≥40)	143,720 (4.4)
**Other reasons for exclusion**	
Receiving end of life care	5055 (0.2)
Housebound	6440 (0.2)
Received COVID-19 vaccine within 90 days prior to campaign start	20,655 (0.6)
Received 3rd COVID-19 vaccine prior to wider availability*	5745 (0.2)
Received 4th COVID-19 vaccine prior to wider availability*	59,660 (1.8)
Did not receive second primary COVID-19 vaccine dose	521,165 (16.0)

^ *n* = 3,254,000 used as the denominator to calculate percentages.

* As a proxy for immunosuppressed individuals who were eligible for a third primary dose.

**Table 2:** Demographic characteristics of people 45–54 years in final cohort on 3 September 2022. All counts rounded to nearest 5.

	45–49 years	50–54 years
	**N (%)**	**N (%)**
**Total population**	906,040	1,011,330
	(100.0)	(100.0)
**Number of previous SARS-CoV-2 vaccine doses**		
2 doses	162,560 (17.9)	126,235 (12.5)
3 doses	743,485 (82.1)	885,095 (87.5)
**Sex**		
Male	465,730 (51.4)	516,310 (51.1)
Female	440,320 (48.6)	495,020 (48.9)
**Index of Multiple Deprivation (IMD) quintile**		
1 (most deprived)	141,430 (15.6)	148,365 (14.7)
2	161,920 (17.9)	177,060 (17.5)
3	188,555 (20.8)	215,485 (21.3)
4	193,865 (21.4)	223,280 (22.1)
5 (least deprived)	195,295 (21.6)	223,155 (22.1)
Missing	24,975 (2.8)	23,985 (2.4)
**Ethnicity**		
White	661,145 (73.0)	776,560 (76.8)
Asian or Asian British	75,345 (8.3)	55,080 (5.4)
Black	21,365 (2.4)	18,760 (1.9)
Mixed	10,335 (1.1)	8395 (0.8)
Other	19,970 (2.2)	15,350 (1.5)
Unknown	117,875 (13.0)	137,175 (13.6)
**Practice region**		
East	219,695 (24.2)	237,570 (23.5)
East Midlands	156,335 (17.3)	179,650 (17.8)
London	62,325 (6.9)	55,830 (5.5)
North East	38,995 (4.3)	46,130 (4.6)
North West	75,125 (8.3)	89,675 (8.9)
South East	60,165 (6.6)	67,150 (6.6)
South West	131,115 (14.5)	152,455 (15.1)
West Midlands	32,595 (3.6)	35,730 (3.5)
Yorkshire and The Humber	125,795 (14.0)	144,355 (14.3)
Missing	2900 (0.3)	2795 (0.3)

**Table 3:** Number of events and risks of primary and secondary outcomes with different index dates by age. All counts rounded to nearest 5.

	Before start of campaign (3 Sep-14 Oct 2022)		At start of campaign (15 Oct-25 Nov 2022)		6 weeks after start of campaign (26 Nov 2022-6 Jan 2023)	
	n	Risk per 100,000	n	Risk per 100,000	n	Risk per 100,000
**No. people**						
45–49 years	906,050	–	904,900	–	903,680	–
50–54 years	1,011,330	–	1,010,735	–	1,010,150	–
**COVID-19 composite (unplanned admission, A&E attendance, or death)**						
45–49 years	105	11.6	110	12.2	115	12.7
50–54 years	155	15.3	105	10.4	145	14.4
**Respiratory composite (unplanned admission or death)**						
45–49 years	190	21.0	275	30.4	470	52.0
50–54 years	215	21.3	300	29.7	540	53.5
**Any unplanned admission**						
45–49 years	3920	432.7	4105	453.6	3710	410.5
50–54 years	4490	444.0	4800	474.9	4515	447.0
**Any death**						
45–49 years	50	5.5	55	6.1	55	6.1
50–54 years	90	8.9	105	10.4	135	13.4

A&E = accident and emergency.

**Table 4:** Estimates from sharp regression discontinuity analysis estimating change in 6-week outcomes at threshold (50 years) using different index dates. Bold indicates primary analysis.

Index date	COVID-19 composite (unplanned admission, A&E attendance or death)	Respiratory composite (admission or death)	Any unplanned admission	Any death
	Estimate (95 % CI)	Estimate (95 % CI)	Estimate (95 % CI)	Estimate (95 % CI)
Control period:	0.6 (–6.1 to 7.4)	0.7 (–6.3 to 7.6)	–13.0 (–49.9 to 23.9)	1.6 (–3.9 to 7.1)
2022-09-03				
2022-10-15	– 5.4 (–12.8 to 1.9)	– 3.7 (–11.9 to 4.6)	5.2 (–29.8 to 40.2)	3.4 (–0.4 to 7.3)
**2022-11-26**	**–0.4 (–7.8 to 7.1)**	**–0.6 (–13.5 to 12.3)**	**5.0 (–40.7 to 50.8)**	**3.0 (–2.7 to 8.6)**
2022-11-27	–1.4 (–8.5 to 5.8)	–1.0 (–13.6 to 11.6)	10.1 (–32.9 to 53.1)	3.3 (–2.4 to 9.1)
2022-11-28	–1.8 (–9.1 to 5.5)	– 2.2 (–14.9 to 10.4)	10.1 (–32.4 to 52.6)	3.5 (–1.7 to 8.7)
2022-11-29	–1.4 (–8.7 to 5.8)	–1.2 (–14.2 to 11.8)	9.9 (–31.6 to 51.3)	2.6 (–2.5 to 7.8)
2022-11-30	–1.0 (–8.1 to 6.1)	– 2.9 (–15.2 to 9.3)	10.5 (–29.6 to 50.6)	3.7 (–1.2 to 8.7)
2022-12-01	– 0.7 (–7.8 to 6.4)	– 2.1 (–14.8 to 10.7)	5.7 (–33.2 to 44.7)	3.4 (–1.5 to 8.3)
2022-12-02	–1.0 (–8.0 to 6.0)	– 2.1 (–15.7 to 11.5)	6.2 (–29.1 to 41.6)	3.9 (–1.2 to 8.9)
2022-12-03	–1.2 (–8.2 to 5.8)	–1.2 (–14.6 to 12.2)	8.5 (–26.6 to 43.7)	3.2 (–1.9 to 8.2)
2022-12-04	–1.2 (–8.3 to 6.0)	–1.7 (–14.9 to 11.4)	10.4 (–23.9 to 44.6)	3.4 (–1.8 to 8.6)
2022-12-05	–1.6 (–8.8 to 5.6)	–1.2 (–14.3 to 11.9)	12.9 (–22.2 to 48.0)	3.4 (–2.0 to 8.8)
2022-12-06	– 0.5 (–7.3 to 6.3)	–1.0 (–13.9 to 11.8)	9.1 (–26.3 to 44.5)	2.3 (–3.2 to 7.9)
2022-12-07	– 0.4 (–7.0 to 6.1)	–1.8 (–14.5 to 10.8)	5.1 (–29.5 to 39.7)	1.9 (–3.4 to 7.3)
2022-12-08	– 0.8 (–7.0 to 5.5)	– 2.6 (–15.5 to 10.4)	10.4 (–24.9 to 45.6)	1.4 (–4.1 to 6.8)
2022-12-09	–1.0 (–6.9 to 4.9)	– 2.3 (–14.7 to 10.0)	7.3 (–27.1 to 41.7)	1.1 (–4.6 to 6.8)

A&E = accident and emergency.

## Data Availability

A link to all code and codelists used in this study is provided in the manuscript. The data cannot be shared as they are confidential.

## References

[1] Polack FP, Thomas SJ, Kitchin N, Absalon J, Gurtman A, Lockhart S (2020). Safety and efficacy of the BNT162b2 mRNA Covid-19 vaccine. N Engl J Med.

[2] Voysey M, Clemens SAC, Madhi SA, Weckx LY, Folegatti PM, Aley PK (2021). Safety and efficacy of the ChAdOx1 nCoV-19 vaccine (AZD1222) against SARS-CoV-2: an interim analysis of four randomised controlled trials in Brazil, South Africa, and the UK. Lancet.

[3] Horne EMF, Hulme WJ, Keogh RH, Palmer TM, Williamson EJ, Parker EPK (2022). Waning effectiveness of BNT162b2 and ChAdOx1 covid-19 vaccines over six months since second dose: OpenSAFELY cohort study using linked electronic health records. BMJ.

[4] Kirsebom FCM, Andrews N, Stowe J, Toffa S, Sachdeva R, Gallagher E (2022). COVID-19 vaccine effectiveness against the omicron (BA.2) variant in England. Lancet Infect Dis.

[5] Andrews N, Stowe J, Kirsebom F, Toffa S, Rickeard T, Gallagher E (2022). Covid-19 vaccine effectiveness against the omicron (B.1.1.529) variant. N Engl J Med.

[6] Stowe J, Andrews N, Kirsebom F, Ramsay M, Bernal JL (2022). Effectiveness of COVID-19 vaccines against omicron and Delta hospitalisation, a test negative case-control study. Nat Commun.

[7] Horne EMF, Hulme WJ, Parker EPK, Keogh RH, Williamson EJ, Walker VM (2024). Effectiveness of mRNA COVID-19 Vaccines as First Booster Doses in England: An Observational Study in OpenSAFELY-TPP. Epidemiology.

[8] Arbel R, Sergienko R, Friger M, Peretz A, Beckenstein T, Yaron S (2022). Effectiveness of a second BNT162b2 booster vaccine against hospitalization and death from COVID-19 in adults aged over 60 years. Nat Med.

[9] Andrews N, Stowe J, Kirsebom F, Toffa S, Sachdeva R, Gower C (2022). Effectiveness of COVID-19 booster vaccines against COVID-19-related symptoms, hospitalization and death in England. Nat Med.

[10] NHS England (2022). NHS COVID-19 vaccine bookings to open to millions of people as autumn booster campaign kicks off.

[11] NHS England (2022). Over 65s can now book autumn COVID booster.

[12] NHS England (2022). NHS invites people 50 and over for autumn boosters and flu jab.

[13] UK Health Security Agency (2023). GOV.UK. COVID-19: the green book, chapter 14a.

[14] Hulme WJ, Horne EMF, Parker EPK, Keogh RH, Williamson EJ, Walker V (2023). Comparative effectiveness of BNT162b2 versus mRNA-1273 covid-19 vaccine boosting in England: matched cohort study in OpenSAFELY-TPP. BMJ.

[15] Imbens GW, Lemieux T (2008). Regression discontinuity designs: a guide to practice. J Econ.

[16] Moscoe E, Bor J, Bärnighausen T (2015). Regression discontinuity designs are underutilized in medicine, epidemiology, and public health: a review of current and best practice. J Clin Epidemiol.

[17] Oldenburg CE, Moscoe E, Bärnighausen T (2016). Regression discontinuity for causal effect estimation in epidemiology. Curr Epidemiol Rep.

[18] Bermingham C, Morgan J, Ayoubkhani D, Glickman M, Islam N, Sheikh A (2023). Estimating the effectiveness of first dose of COVID-19 vaccine against mortality in England: a quasi-experimental study. Am J Epidemiol.

[19] Anderson ML, Dobkin C, Gorry D (2020). The effect of influenza vaccination for the elderly on hospitalization and mortality. Ann Intern Med.

[20] Smith LM, Lévesque LE, Kaufman JS, Strumpf EC (2017). Strategies for evaluating the assumptions of the regression discontinuity design: a case study using a human papillomavirus vaccination programme. Int J Epidemiol.

[21] Hernán MA, Robins JM (2020). Causal inference: What if.

[22] Bar-On YM, Goldberg Y, Mandel M, Bodenheimer O, Amir O, Freedman L (2022). Protection by a fourth dose of BNT162b2 against omicron in Israel. N Engl J Med.

[23] Mateo-Urdiales A, Sacco C, Fotakis EA, Manso MD, Bella A, Riccardo F (2023). Relative effectiveness of monovalent and bivalent mRNA boosters in preventing severe COVID-19 due to omicron BA.5 infection up to 4 months post-administration in people aged 60 years or older in Italy: a retrospective matched cohort study. Lancet Infect Dis.

[24] Lin DY, Xu Y, Gu Y, Zeng D, Wheeler B, Young H (2023). Effectiveness of bivalent boosters against severe omicron infection. N Engl J Med.

[25] Mendes D, Machira Krishnan S, O’Brien E, Padgett T, Harrison C, Strain WD (2024). Modelling COVID-19 vaccination in the UK: impact of the autumn 2022 and spring 2023 booster campaigns. Infect Dis Ther.

[26] Kirsebom FCM, Andrews N, Stowe J, Ramsay M, Bernal JL (2023). Duration of protection of ancestral-strain monovalent vaccines and effectiveness of bivalent BA.1 boosters against COVID-19 hospitalisation in England: a test-negative case-control study. Lancet Infect Dis.

[27] Andersson NW, Thiesson EM, Baum U, Pihlström N, Starrfelt J, Faksová K (2023). Comparative effectiveness of bivalent BA.4-5 and BA.1 mRNA booster vaccines among adults aged ≥ 50 years in Nordic countries: nationwide cohort study. BMJ.

[28] Wee LE, Pang D, Chiew C, Tan J, Lee V, Ong B (2023). Long-term real-world protection afforded by third mRNA doses against symptomatic SARS-COV-2 infections, COVID-19-related emergency attendances and hospitalizations amongst older Singaporeans during an omicron XBB wave. Clin Infect Dis.

[29] Office for National Statistics (2023). Coronavirus (COVID-19) latest insights.

[30] Thompson EJ, Williams DM, Walker AJ, Mitchell RE, Niedzwiedz CL, Yang TC (2022). Long COVID burden and risk factors in 10 UK longitudinal studies and electronic health records. Nat Commun.

[31] Schaffer AL, Park RY, Tazare J, Bhaskaran K, MacKenna B, Denaxas S (2024). Changes in sick notes associated with COVID-19 from 2020 to 2022: A cohort study in 24 million primary care patients in OpenSAFELY-TPP. BMJ Open.

[32] Griffith GJ, Morris TT, Tudball MJ, Herbert A, Mancano G, Pike L (2020). Collider bias undermines our understanding of COVID-19 disease risk and severity. Nat Commun.

[33] Benjamin-Chung J, Arnold BF, Berger D, Luby SP, Miguel E, Colford JM (2018). Spillover effects in epidemiology: parameters, study designs and methodological considerations. Int J Epidemiol.

[34] Hause AM (2022). Safety Monitoring of Bivalent COVID-19 mRNA Vaccine Booster Doses Among Persons Aged ≥12 Years — United StatesAugust 31–October 23; 2022. MMWR Morb Mortal Wkly Rep.

[35] World Health Organisation Strategic Advisory Group of Experts on Immunisation (2023). SAGE updates COVID-19 vaccination guidance.

[36] Andrews C, Schultze A, Curtis H, Hulme W, Tazare J, Evans S (2022). OpenSAFELY: representativeness of electronic health record platform OpenSAFELY-TPP data compared to the population of England. Wellcome Open Res.

[37] Hulme WJ, Williamson E, Horne EMF, Green A, McDonald HI, Walker AJ (2023). Challenges in estimating the effectiveness of COVID-19 vaccination using observational data. Ann Intern Med.

