# Supplementary material for: Effect of the 2022 COVID-19 booster vaccination campaign in people aged 50 years in England: Regression discontinuity analysis in OpenSAFELY-TPP
Source: Vaccine. Author manuscript; Available in PMC 2026 Jan 29. (PMC7618680; doi:10.1016/j.vaccine.2025.127257)
Supplement: Supplementary material [file EMS212183-supplement-Supplementary_material.pdf]

Supplementary files

Supplementary Table 1. List of exclusion criteria and their definition. Unless otherwise stated, all criteria were identified using primary care data. Codelists with all codes used to define criteria are available in the GitHub repository: https://github.com/opensafely/vax-fourth-dose-RD

Exclusion criteria		Definition
JCVI high risk groups prioritised for vaccination(1)	Chronic respiratory disease (including poorly controlled asthma)	- Chronic respiratory disease diagnosis any time prior to index date;<br>- Asthma admission recorded in primary care any time prior to index date; OR<br>- Asthma diagnosis any time prior to index date with a prescription for systemic steroids in each of the 3 months prior to the index date.
	Chronic heart disease and vascular disease	Diagnosis any time prior to index date
	Chronic kidney disease	Diagnosis any time prior to index date
	Chronic liver disease	Diagnosis any time prior to index date
	Chronic neurological disease	Diagnosis any time prior to index date
	Learning disability	Diagnosis any time prior to index date
	Diabetes mellitus and other endocrine disorders	Diagnosis any time prior to index date, either in the absence, or occurring after, a resolved diabetes code

	Immunosuppressed (including HIV/AIDS)	- Diagnosis of condition causing immunosuppression (including HIV infection/AIDS or solid organ transplant) any time prior to index date; OR<br>- Cancer diagnosis anytime in 3 years prior to index date; OR<br>- Prescription for a chemotherapeutic, immunosuppressant, or immunomodulating medicine in 6 months prior to the index date
	Morbid obesity	- Diagnosis of severe obesity (BMI >=40) following date of BMI being recorded; OR<br>- Most recent recorded BMI value >=40
	Asplenia	Diagnosis any time prior to index date
	Severe mental illness	Diagnosis any time prior to index date, either in the absence, or occurring after, a severe mental illness in remission code
Receipt of third or fourth booster dose prior to first availability to non-immunosuppressed population		Evidence of a third COVID-19 vaccination prior to 16 September 2021(2), or a fourth COVID-19 vaccination prior to 5 September 2022(3)
Health or social care worker		Stated that they were a health or social care worker when receiving at least one of their COVID-19 vaccinations
End of life		- Code indicating end of life recorded in primary care; OR<br>- Prescription for midazolam injection indicated for treatment of pain at end of life any time prior to index date.
Residents in a care or nursing home		- Care home residence code any time prior to index date; OR<br>- Current address maps to list of care homes(4)

Housebound people	- Code indicating person is housebound, in absence of: a code indicating that the person is no longer housebound; a code indicating the person is in a care home

(1) UK Health Security Agency. GOV.UK. 2023 [cited 2023 Mar 10]. COVID-19: the green book, chapter 14a. Available from: https://www.gov.uk/government/publications/covid-19-the-green-book-chapter-14a

(2) NHS England. NHS begins COVID-19 booster vaccination campaign [Internet]. [cited 2023 Mar 17]. Available from: https://www.england.nhs.uk/2021/09/nhs-begins-covid-19-booster-vaccination-campaign/

(3) NHS England. NHS invites people 50 and over for autumn boosters and flu jab [Internet]. 2022 [cited 2023 Mar 10]. Available from: https://www.england.nhs.uk/2022/10/nhs-invites-people-50-and-over-for-autumn-boosters-and-flu-jab/

(4) Schultze A, Bates C, Cockburn J, MacKenna B, Nightingale E, Curtis HJ, et al. Identifying Care Home Residents in Electronic Health Records - An OpenSAFELY Short Data Report. Wellcome Open Res. 2021;(6):90.

Supplementary Table 2. List of codes used to define outcomes.

Outcome	Codelist
**COVID-19 hospital admission**	Unplanned hospital admission with any of the following ICD-10 codes in any position (primary or secondary): U071, U072, U099, U109
**COVID-19 emergency attendance**	Emergency attendances with any of the following SNOMED codes: 1240751000000100, 1325161000000102, 1325171000000109, 132581000000106
**COVID-19 death**	Deaths with any of the following ICD-10 codes on the death certificate in any position (underlying or contributing): U071, U072, U099, U109
**Respiratory admission**	Unplanned hospital admission with any of the following ICD-10 codes in the primary position (J00-J99)
**Respiratory death**	Deaths with any of the following ICD-10 codes on the death certificate (underlying only): (J00-J99)

Supplementary Figure 1. Sex relative frequency distribution by age in 3-month intervals based on age at 3 September 2022.

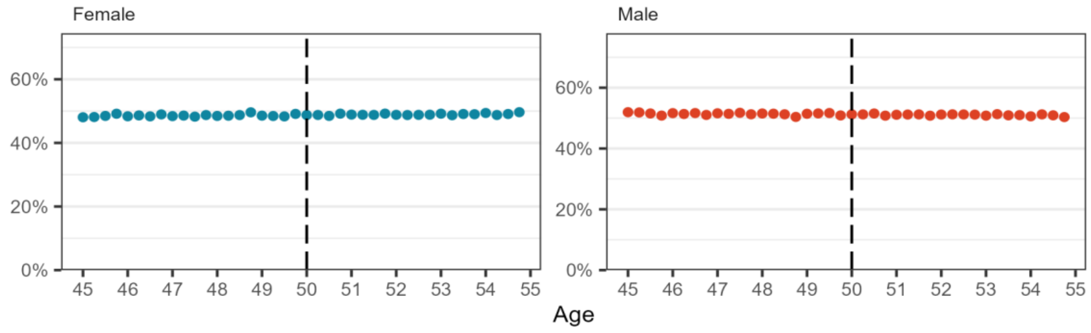

Supplementary Figure 2. IMD relative frequency distribution by age in 3-month intervals based on age at 3 September 2022

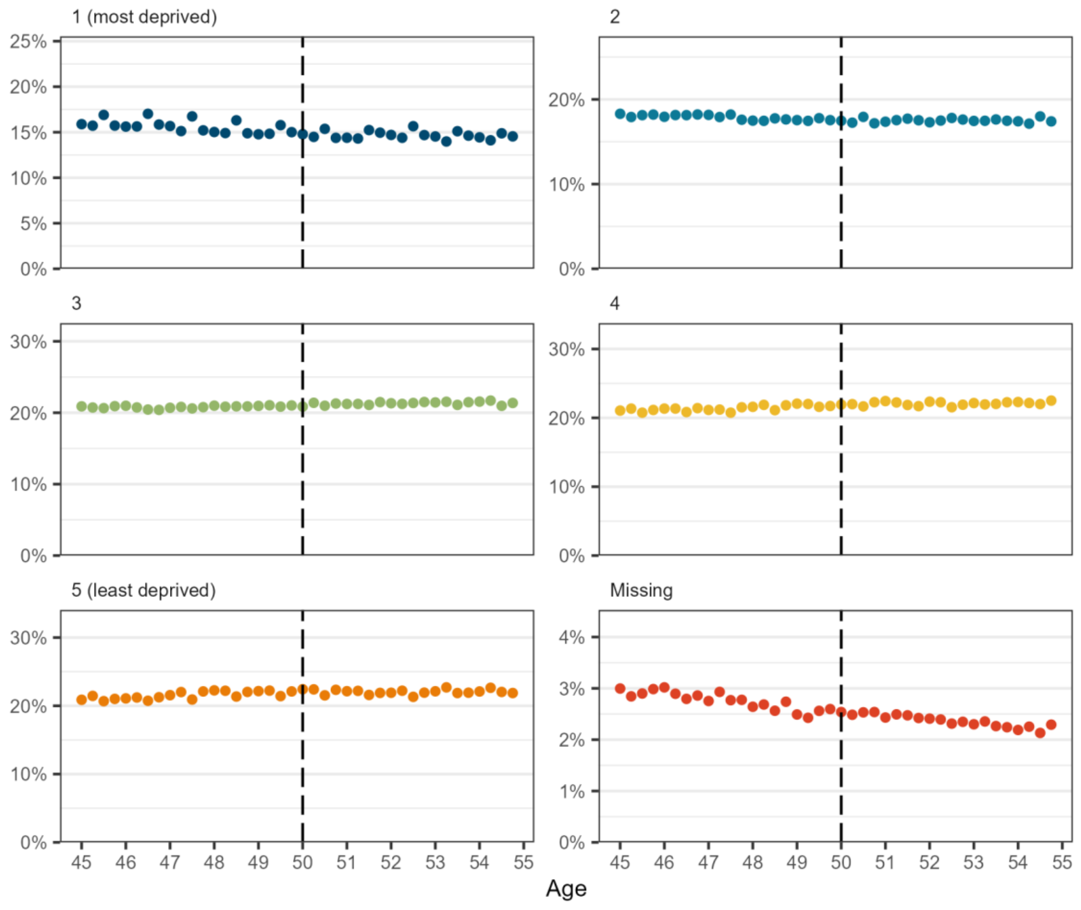

Supplementary Figure 3. Ethnicity relative frequency distribution by age in 3-month intervals based on age at 3 September 2022

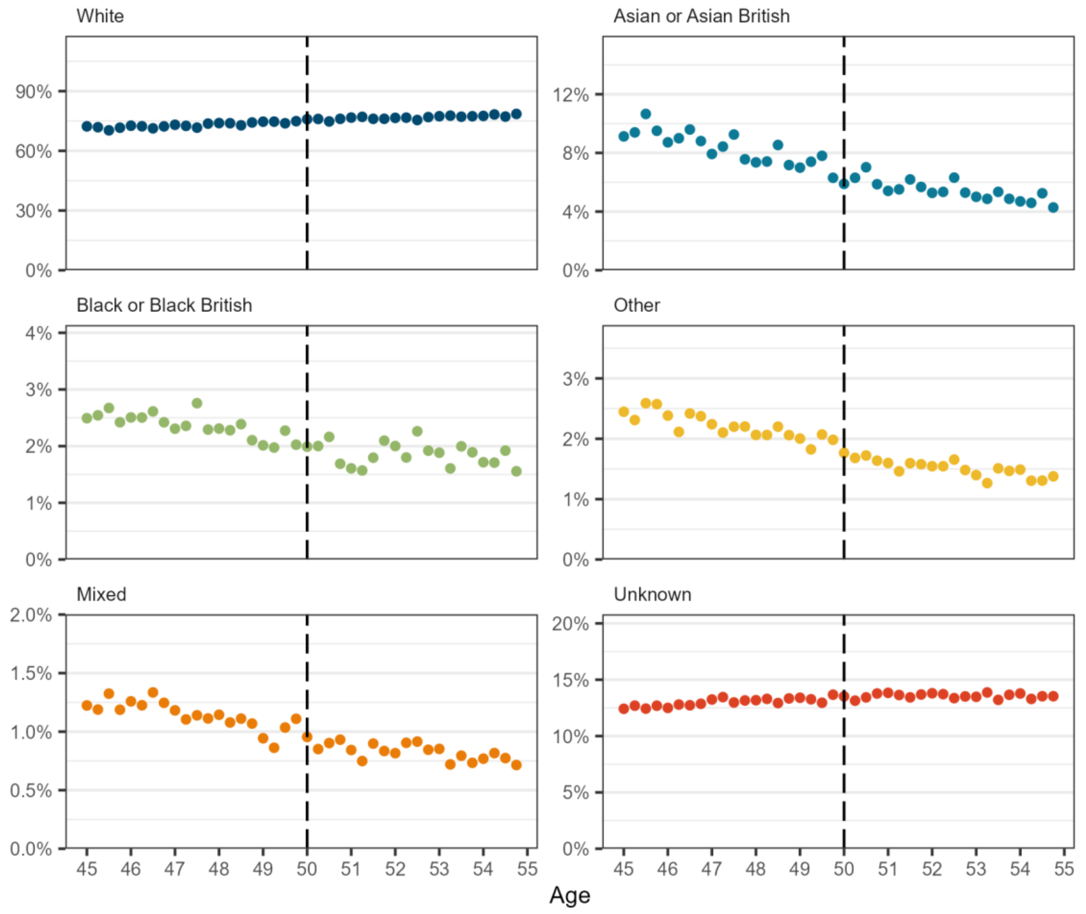

Supplementary Figure 4. Region relative frequency distribution by age in 3-month intervals based on age at 3 September 2022

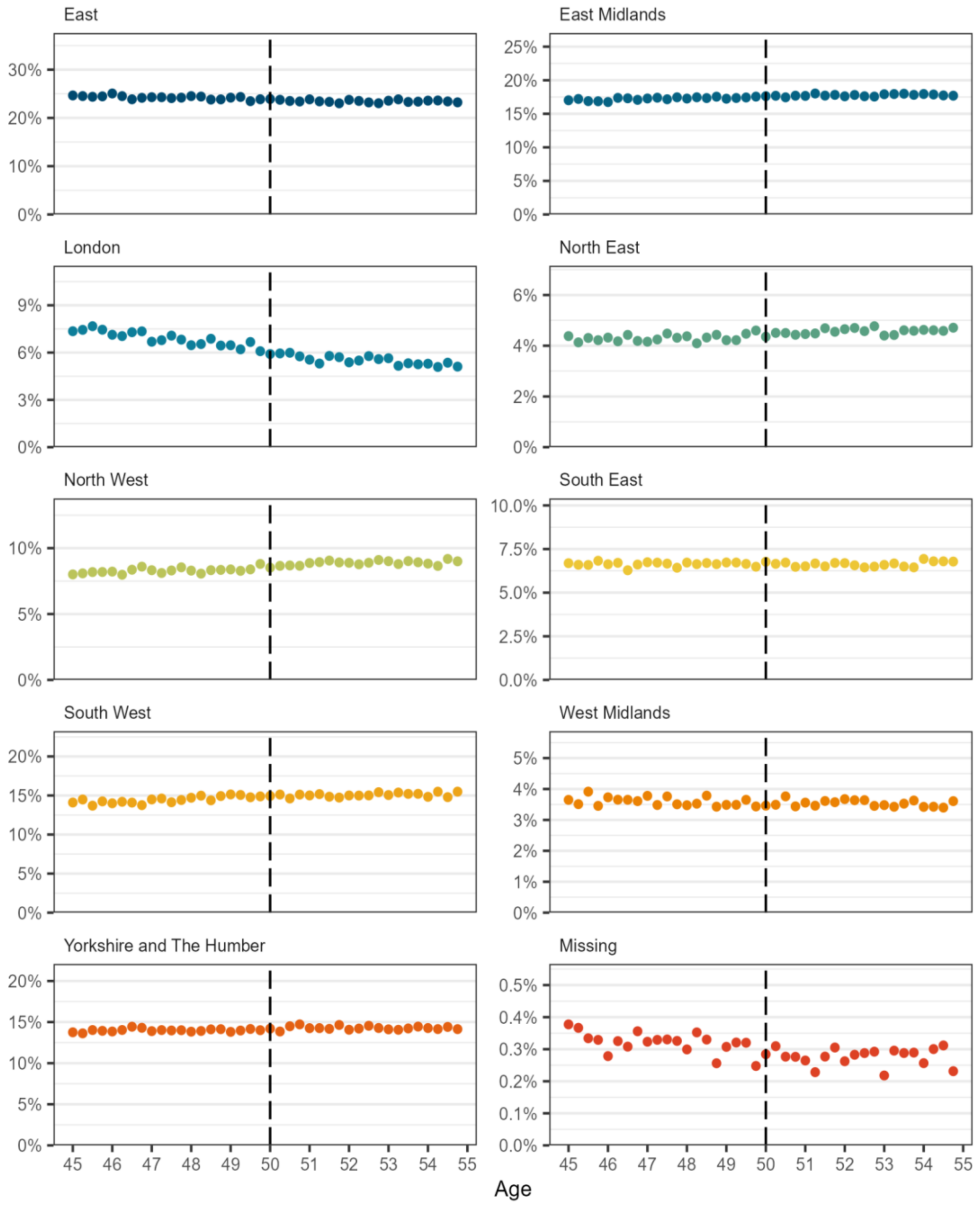

Supplementary Figure 5. Relative frequency distribution of previous number of COVID-19 vaccine doses by age in 3-month intervals based on age at 3 September 2022

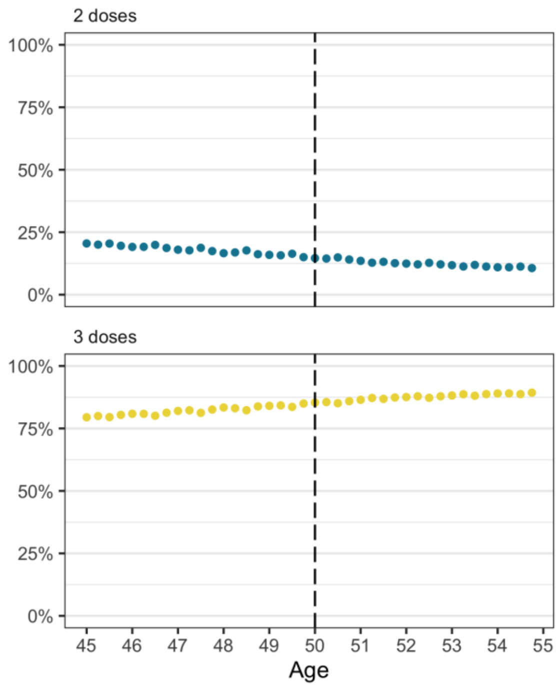

Supplementary Figure 6. Coverage of 2022/23 influenza vaccination by 26 November 2023 by age in 3-month intervals

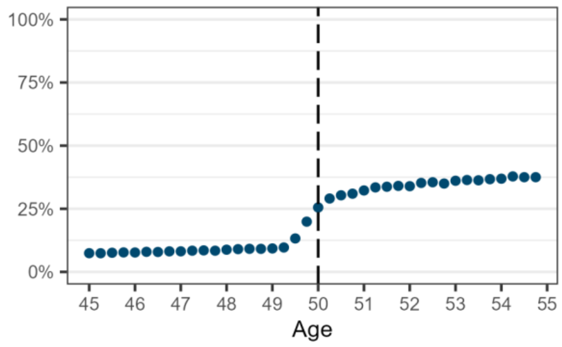

Supplementary Table 3. Estimates from sharp regression discontinuity analysis excluding people born in the index month, estimating change in 6-week outcomes at threshold (50 years) using different index dates. Bold indicates primary analysis.

	**COVID-19 composite (unplanned admission, A&E attendance or death)**	**Respiratory composite (admission or death)**	**Any unplanned admission**	**Any death**
**Index date**	**Estimate (95% CI)**	**Estimate (95% CI)**	**Estimate (95% CI)**	**Estimate (95% CI)**
Control period: 2022-09-03	1.3 (-5.9 to 8.4)	-2.1 (-8.8 to 4.5)	-16.0 (-55.1 to 23.1)	2.5 (-3.2 to 8.3)
2022-10-15	-5.8 (-13.6 to 2.0)	-4.8 (-13.4 to 3.9)	1.3 (-35.7 to 38.3)	2.7 (-1.4 to 6.7)
**2022-11-26**	**-1.8 (-9.5 to 6.0)**	**-2.8 (-16.2 to 10.7)**	**-3.6 (-51.1 to 43.9)**	**4.6 (-1.1 to 10.3)**
2022-11-27	-2.8 (-10.2 to 4.6)	-3.2 (-16.3 to 9.9)	2.4 (-42.4 to 47.1)	4.9 (-0.9 to 10.8)
2022-11-28	-3.2 (-10.8 to 4.3)	-4.2 (-17.4 to 9.0)	1.3 (-42.6 to 45.2)	5.2 (0.0 to 10.4)
2022-11-29	-3.2 (-10.7 to 4.2)	-2.5 (-16.2 to 11.2)	0.9 (-41.8 to 43.6)	4.2 (-0.9 to 9.4)
2022-11-30	-2.8 (-10.0 to 4.5)	-4.3 (-17.2 to 8.7)	2.6 (-38.9 to 44.2)	5.1 (0.0 to 10.1)
2022-12-01	-2.5 (-9.7 to 4.8)	-3.7 (-17.1 to 9.7)	-3.4 (-43.3 to 36.6)	4.7 (-0.3 to 9.6)
2022-12-02	-2.8 (-10.0 to 4.3)	-3.9 (-18.2 to 10.4)	-0.5 (-37.2 to 36.2)	5.2 (0.0 to 10.3)
2022-12-03	-3.0 (-10.2 to 4.1)	-3.1 (-17.2 to 11.1)	2.0 (-34.5 to 38.6)	4.8 (-0.1 to 9.8)
2022-12-04	-3.1 (-10.4 to 4.1)	-3.7 (-17.4 to 10.1)	3.5 (-32.0 to 38.9)	5.1 (-0.1 to 10.2)
2022-12-05	-3.6 (-10.9 to 3.7)	-3.6 (-17.2 to 10.0)	5.2 (-30.9 to 41.4)	5.1 (-0.3 to 10.4)
2022-12-06	-2.4 (-9.3 to 4.4)	-2.4 (-15.9 to 11.2)	2.3 (-34.4 to 39.1)	3.9 (-1.6 to 9.5)
2022-12-07	-2.4 (-8.9 to 4.1)	-2.2 (-15.7 to 11.2)	-0.9 (-37.0 to 35.1)	3.5 (-1.8 to 8.8)
2022-12-08	-2.3 (-8.7 to 4.1)	-2.1 (-15.8 to 11.7)	2.6 (-33.7 to 38.9)	2.9 (-2.5 to 8.4)
2022-12-09	-2.2 (-8.3 to 3.9)	-1.5 (-14.5 to 11.6)	-1.1 (-36.2 to 34.0)	2.6 (-3.2 to 8.3)

A&E = accident and emergency

Supplementary Table 4. Estimates from sharp regression discontinuity analysis estimating change in 6-week outcomes at threshold (50 years) using different bandwidths for primary index date (26 November 2022).

	**COVID-19 composite (unplanned admission, A&E attendance or death)**	**Respiratory composite (admission or death)**	**Any unplanned admission**	**Any death**
**Bandwidth**	**Estimate (95% CI)**	**Estimate (95% CI)**	**Estimate (95% CI)**	**Estimate (95% CI)**
4 years	1.0 (-7.0 to 9.1)	2.4 (-12.3 to 17.1)	30.0 (-22.3 to 82.2)	1.3 (-4.7 to 7.3)
3 years	-0.8 (-10.4 to 8.7)	2.9 (-15.5 to 21.3)	16.5 (-42.7 to 75.7)	1.9 (-6.3 to 10.0)
2 years	1.9 (-9.5 to 13.3)	2.2 (-19.7 to 24.0)	16.3 (-62.9 to 95.5)	-5.9 (-15.7 to 3.8)
1 year	2.0 (-17.9 to 21.9)	12.2 (-32.1 to 56.5)	13.0 (-186.9 to 212.8)	-9.6 (-27.8 to 8.5)

A&E = accident and emergency

Supplementary Table 5. Instrumental variable analysis (fuzzy regression discontinuity) estimating local average treatment effect (LATE) for 6-week outcomes with different index dates. Bold indicates primary analysis.

	**COVID-19 composite (unplanned admission, A&E attendance or death)**	**Respiratory composite (admission or death)**	**Any unplanned admission**	**Any death**
**Index date**	**Estimate (95% CI)**	**Estimate (95% CI)**	**Estimate (95% CI)**	**Estimate (95% CI)**
**2022-11-26**	**2.1 (-11.3 to 15.4)**	**-12.0 (-58.2 to 34.3)**	**15.4 (-166.7 to 197.6)**	**14.7 (-6.4 to 35.8)**
2022-11-27	-1.4 (-14.4 to 11.7)	-12.3 (-54.4 to 29.7)	32.8 (-128.0 to 193.5)	16.0 (-3.6 to 35.6)
2022-11-28	-2.8 (-16.3 to 10.8)	-17.1 (-58.6 to 24.4)	30.9 (-130.1 to 191.8)	16.7 (-1.6 to 35.0)
2022-11-29	-4.4 (-27.6 to 18.8)	-4.7 (-48.3 to 38.9)	29.6 (-120.2 to 179.3)	9.5 (-10.8 to 29.7)
2022-11-30	-2.7 (-24.6 to 19.1)	-10.4 (-51.3 to 30.4)	31.8 (-112.4 to 176.0)	13.2 (-6.3 to 32.7)
2022-12-01	-2.4 (-25.2 to 20.3)	-6.9 (-54.5 to 40.6)	19.7 (-125.6 to 165.0)	11.5 (-8.5 to 31.5)
2022-12-02	-3.5 (-26.7 to 19.7)	-7.1 (-57.9 to 43.6)	21.1 (-118.4 to 160.6)	13.0 (-7.8 to 33.8)
2022-12-03	-3.9 (-27.1 to 19.2)	-4.0 (-54.7 to 46.7)	28.6 (-102.9 to 160.1)	10.6 (-10.6 to 31.8)
2022-12-04	-3.8 (-29.2 to 21.5)	-5.7 (-55.6 to 44.2)	34.4 (-99.2 to 168.0)	11.3 (-9.3 to 31.8)
2022-12-05	-5.3 (-30.7 to 20.1)	-3.9 (-54.1 to 46.4)	42.6 (-92.1 to 177.3)	11.1 (-9.7 to 31.9)
2022-12-06	-1.7 (-25.1 to 21.7)	-3.4 (-51.3 to 44.5)	29.9 (-106.1 to 165.9)	7.6 (-12.9 to 28.1)
2022-12-07	-1.5 (-24.5 to 21.6)	-6.0 (-51.3 to 39.4)	16.8 (-107.1 to 140.8)	6.3 (-12.9 to 25.5)
2022-12-08	-2.5 (-24.2 to 19.2)	-8.3 (-55.3 to 38.7)	33.7 (-97.9 to 165.2)	4.4 (-15.3 to 24.1)
2022-12-09	-3.3 (-23.4 to 16.8)	-7.5 (-55.0 to 40.1)	23.7 (-108.7 to 156.2)	3.5 (-16.4 to 23.4)

A&E = accident and emergency

Supplementary Table 6. Instrumental variable analysis (fuzzy regression discontinuity) estimating local average treatment effect (LATE) for 6-week outcomes with different index dates including receipt of influenza vaccination in the model. Bold indicates primary analysis.

	**COVID-19 composite (unplanned admission, A&E attendance or death)**	**Respiratory composite (admission or death)**	**Any unplanned admission**	**Any death**
**Index date**	**Estimate (95% CI)**	**Estimate (95% CI)**	**Estimate (95% CI)**	**Estimate (95% CI)**
**2022-11-26**	**5.4 (-24.9 to 35.8)**	**-24.7 (-153.9 to 104.6)**	**8.3 (-286.4 to 303.1)**	**27.1 (-10.5 to 64.8)**
2022-11-27	0.8 (-30.1 to 31.7)	-24.1 (-145.9 to 97.8)	36.3 (-251.8 to 324.4)	28.6 (-7.3 to 64.5)
2022-11-28	-1.7 (-39.0 to 35.7)	-29.9 (-148.0 to 88.1)	29.5 (-267.6 to 326.6)	29.7 (-7.4 to 66.9)
2022-11-29	-4.0 (-47.1 to 39.1)	-14.9 (-131.7 to 101.9)	24.3 (-268.2 to 316.8)	19.7 (-28.6 to 68.1)
2022-11-30	-2.0 (-42.7 to 38.7)	-24.1 (-154.9 to 106.7)	26.8 (-281 to 334.5)	25.1 (-22.3 to 72.4)
2022-12-01	-2.0 (-44.6 to 40.6)	-19.2 (-157.9 to 119.5)	5.7 (-298.2 to 309.5)	22.7 (-18.2 to 63.6)
2022-12-02	-3.5 (-45.3 to 38.2)	-18.2 (-159.4 to 123.0)	8.5 (-283.7 to 300.6)	24.9 (-17.3 to 67.1)
2022-12-03	-4.1 (-45.2 to 36.9)	-15.0 (-156.6 to 126.5)	13.0 (-263.3 to 289.2)	21.3 (-17.3 to 59.8)
2022-12-04	-4.2 (-47.1 to 38.7)	-17.5 (-164.1 to 129.1)	22.6 (-272.7 to 317.9)	22.1 (-13.8 to 58.0)
2022-12-05	-6.8 (-48.9 to 35.3)	-15.0 (-163.3 to 133.3)	31.0 (-265.8 to 327.8)	22.1 (-14.4 to 58.5)
2022-12-06	-1.9 (-43.5 to 39.7)	-13.5 (-162.2 to 135.1)	14.0 (-278.9 to 306.9)	16.9 (-18.0 to 51.7)
2022-12-07	-2.1 (-42.1 to 37.9)	-17.0 (-163.8 to 129.7)	-7.9 (-293.8 to 277.9)	14.9 (-17.9 to 47.7)
2022-12-08	-3.5 (-41.7 to 34.7)	-19.1 (-163.2 to 125.0)	16.4 (-291.5 to 324.3)	12.1 (-20.3 to 44.5)
2022-12-09	-4.6 (-40.0 to 30.8)	-17.6 (-157.4 to 122.1)	2.7 (-308.6 to 314.1)	10.9 (-21.4 to 43.1)

A&E = accident and emergency

Supplementary Table 7. Estimates from fuzzy regression discontinuity analysis estimating change in 6-week outcomes at threshold (50 years) using different bandwidths for primary index date (26 November 2022).

	**COVID-19 composite (unplanned admission, A&E attendance or death)**	**Respiratory composite (admission or death)**	**Any unplanned admission**	**Any death**
**Bandwidth**	**Estimate (95% CI)**	**Estimate (95% CI)**	**Estimate (95% CI)**	**Estimate (95% CI)**
4 years	5.5 (-9.9 to 21.0)	-0.8 (-52.8 to 51.2)	92.0 (-128.0 to 311.9)	12.6 (-12.4 to 37.7)
3 years	-5.7 (-26.6 to 15.3)	-3.0 (-69.3 to 63.3)	51.2 (-218 to 320.4)	10.4 (-18.9 to 39.7)
2 years	0.6 (-22.0 to 23.2)	-1.0 (-75.5 to 73.4)	38.6 (-290.2 to 367.5)	-12.0 (-46.7 to 22.8)
1 year	5.5 (-9.9 to 21.0)	-0.8 (-52.8 to 51.2)	92.0 (-128.0 to 311.9)	12.6 (-12.4 to 37.7)

A&E = accident and emergency

Information governance

NHS England is the data controller of the NHS England OpenSAFELY COVID-19 Service; TPP is the data processor; all study authors using OpenSAFELY have the approval of NHS England.(1) This implementation of OpenSAFELY is hosted within the TPP environment which is accredited to the ISO 27001 information security standard and is NHS IG Toolkit compliant.(2)

Patient data has been pseudonymised for analysis and linkage using industry standard cryptographic hashing techniques; all pseudonymised datasets transmitted for linkage onto OpenSAFELY are encrypted; access to the NHS England OpenSAFELY COVID-19 service is via a virtual private network (VPN) connection; the researchers hold contracts with NHS England and only access the platform to initiate database queries and statistical models; all database activity is logged; only aggregate statistical outputs leave the platform environment following best practice for anonymisation of results such as statistical disclosure control for low cell counts.(3)

The service adheres to the obligations of the UK General Data Protection Regulation (UK GDPR) and the Data Protection Act 2018. The service previously operated under notices initially issued in February 2020 by the Secretary of State under Regulation 3(4) of the Health Service (Control of Patient Information) Regulations 2002 (COPI Regulations), which required organisations to process confidential patient information for COVID-19 purposes; this set aside the requirement for patient consent.(4) As of 1 July 2023, the Secretary of State has requested that NHS England continue to operate the Service under the COVID-19 Directions 2020.(5) In some cases of data sharing, the common law duty of confidence is met using, for example, patient consent or support from the Health Research Authority Confidentiality Advisory Group.(6)

Taken together, these provide the legal bases to link patient datasets using the service. GP practices, which provide access to the primary care data, are required to share relevant health information to support the public health response to the pandemic, and have been informed of how the service operates.

(1) NHS Digital. The NHS England OpenSAFELY COVID-19 service - privacy notice [Internet]. 2023 [cited 2023 Jul 5]. Available from: https://digital.nhs.uk/coronavirus/coronavirus-covid-19-response-information-governance-hub/the-nhs-england-opensafely-covid-19-service-privacy-notice

(2) NHS Digital. NHS Digital. 2023 [cited 2023 Jul 5]. Data Security and Protection Toolkit. Available from: https://digital.nhs.uk/data-and-information/looking-after-information/data-security-and-information-governance/data-security-and-protection-toolkit

(3) NHS Digital [Internet]. [cited 2023 Mar 6]. ISB1523: Anonymisation Standard for Publishing Health and Social Care Data. Available from: https://digital.nhs.uk/data-and-information/information-standards/information-standards-and-data-collections-including-extractions/publications-and-notifications/standards-and-collections/isb1523-anonymisation-standard-for-publishing-health-and-social-care-data

(4) UK Department of Health and Social Care. GOV.UK. 2022 [cited 2023 Jul 5]. [Withdrawn] Coronavirus (COVID-19): notice under regulation 3(4) of the Health Service (Control of Patient Information) Regulations 2002 – general. Available from: https://www.gov.uk/government/publications/coronavirus-covid-19-notification-of-data-controllers-to-share-information/coronavirus-covid-19-notice-under-regulation-34-of-the-health-service-control-of-patient-information-regulations-2002-general--2

(5) NHS Digital. NHS Digital. 2022 [cited 2023 Jul 5]. Secretary of State for Health and Social Care: COVID-19 Public Health Directions 2020. Available from: https://digital.nhs.uk/about-nhs-digital/corporate-information-and-documents/directions-and-data-provision-notices/secretary-of-state-directions/covid-19-public-health-directions-2020

(6) NHS Health Research Authority. Health Research Authority. [cited 2023 Jul 5]. Confidentiality Advisory Group. Available from: https://www.hra.nhs.uk/about-us/committees-and-services/confidentiality-advisory-group/
